# Early Inflammatory Response following Traumatic Brain Injury in Rabbits Using USPIO- and Gd-Enhanced MRI

**DOI:** 10.1155/2016/8431987

**Published:** 2016-10-27

**Authors:** Lin Ouyang, Si Zeng, Gang Zheng, Guang Ming Lu

**Affiliations:** ^1^Department of Medical Imaging, Southeast Hospital, Medical College of Xiamen University, Xiamen, China; ^2^Department of Medical Imaging, Jinling Hospital, Medical School of Nanjing University, Nanjing, China; ^3^College of Civil Aviation, Nanjing University of Aeronautics and Astronautics, Nanjing, China

## Abstract

*Purpose*. To monitor the inflammatory response (IR) following traumatic brain injury (TBI) before and after the rehabilitation of the blood-brain barrier (BBB) in rabbits using USPIO- and Gd-enhanced MRI.* Materials and Methods*. Twenty white big-eared rabbits with mild TBI (mTBI) were randomly and equally divided into four groups. Rabbits were sacrificed for the brain specimens immediately after the last MRI-monitoring. Sequences were tse-T1WI, tse-T2WI, Gd-T1WI, and USPIO-T1WI. Dynamical MRI presentations were evaluated and compared with pathological findings for each group.* Results*. Twenty-four hours after injury, all rabbits displayed high signal foci on T2WI, while only 55% lesions could be found on Gd-T1WI and none on USPIO-T1WI. The lesions were enhanced on Gd-T1WI in 100% subjects after 48 h and the enhancement sizes augmented to the largest after 72 h. At the time point of 72 h after TBI, 90% lesions were enhanced by USPIO. Five days after injury, 19 lesions showed decreased Gd-enhancement and one disappeared; however, USPIO-enhancement became larger than before. Pathological findings showed microglias slightly appeared in dense leukocytes at 48 h, but became the dominant inflammatory cells after five days.* Conclusions*. Dynamic IR following injury could be monitored by combination of Gd- and USPIO-MRI in mTBI rabbits.

## 1. Introduction

Traumatic brain injury (TBI) is thought to be triggered by a single event; however, patients usually show a highly variable outcome even from the same initiator event [1]. Patient's condition may deteriorate many years after TBI and eventually develop unexpected consequences such as posttraumatic syndrome [2, 3]. The secondary injury of TBI predominally did harm to the brain [4]. The inflammatory response (IR) following TBI is considered to be an important contributory factor [5, 6].

In vivo, early diagnosis of post-TBI IR is important for clinical therapeutic strategies. However, the primary pathophysiology of IR is not well understood. Currently, major method of monitoring post-TBI IR is based on the inflammatory cytokines, such as IL-1, IL-6, IL-10, and TNF-*α* either in serum or cerebrospinal fluid [7, 8]. Also, flow cytometry, immunohistochemistry [9, 10], fast freezing RNA, protein analysis, and immunofluorescence could be used to study the morphology and phenotype of microglia and macrophage [11–14]. However, inflammatory cytokines or cells used as biomarkers for diagnosis of post-TBI IR were still not widely used because of their low accuracy, lack of specificity, and intuition [15]. In recent years, MRI has been a dominant method to diagnose complicated TBI [16, 17]. However, less has been done on animals to apply MRI methods for assessing post-TBI IR [18].

Biological nuclear magnetic imaging has vast perspectives. USPIO (Ultrasmall Superparamagnetic Iron Oxide), consumed by macrophage and free of BBB (blood brain barrier), may be an appropriate tracer. In this paper, we aimed to investigate the changes of early IR following TBI before and after the rehabilitation of the blood-brain barrier (BBB) in rabbits using USPIO- and Gd-enhanced MRI combined with pathological findings.

## 2. Methods and Materials

### 2.1. Animals

Twenty Japanese white big-eared rabbits were obtained from Qingdao Kangda biology technology limited company, Qingdao, China (license number: SCXK (Lu) 2011 0001). Animals were housed in SPF level environment with temperature 20–25°C, humidity 60–65%, and illumination intensity 100–200 lux. Experimental procedures were in strict accordance with the* Guidance Suggestions for the Care and Use of Laboratory Animals*, issued by the Ministry of Science and Technology of China [19]. The rabbits were 10 months old and approximately weighed 2.5 kg.

### 2.2. Controlled Mild TBI (mTBI) Models Establishment and Subgroup

Animal models of TBI were developed in the animal experimental center of the Clinical Southeast Hospital of the Medical College of Xiamen University. Animals were anesthetized with veterinary Sumianxin II (0.1–0.2 mL/kg, Jilin Huamu Animal Health Product, Jilin Province, China) via gluteal muscle injection and pronely fixed on the animal operating table. The right cranial parietal was chosen as the operative entrance border after shaving and sterilizing the skin. A 0.25 cm^2^ area of cranium was removed by craniotomy using a special bone drill, and an aperture through the skull was built with the dura being intact. The aperture was 5 mm right laterally and 5 mm posteriorly from the bregma. A sterile fine needle (14G) perpendicularly pricked the aperture 10 mm deep into the subcortical encephalic parenchyma. Then the needle was moved out and the skin was sutured after disinfection of incision. The injured animal presented a transient limb tic and returned to an consciousness state from anesthesia status 20 minutes after Sumianxin injection.

### 2.3. MRI Examination and Images Evaluations

Twenty mTBI rabbit models with unilateral parietal controlled subcortex injury were randomly equally grouped by monitoring time, which were set at the time points of 24 h, 48 h, 72 h, and 5 d after TBI. Each group included five rabbits. The first group was monitored by MRI at the 24 h after TBI; the second group was, respectively, scanned at 24 h and 48 h after TBI; the third group had MRI, respectively, at the 24 h, 48 h, and 72 h after TBI; and the fourth group was examined at the 24 h, 48 h, 72 h, and 5 d after TBI, respectively. Animals were fixed by prone position and scanned with knee-defined staged-array coil. A 3.0T MR (Magnetom Verio; Siemens, Berlin, German) was used for the MRI monitory. The protocols were shown in Table 1. Contrast agents were administered through rabbit ear vein with a dose of 0.1 mmol/kg (gadolinium diethylene triamine penta-acetic acid, Gd-DTPA; license number: 20080064; producer: Shanghai Bracco Sine Pharmaceutical Corp. Ltd.). USPIO (provided by Molecular Laboratory of Medicine Image Center of Jinling Hospital, Nanjing) was given at a dosage of 0.05 mmol/kg (concentration 1.2 mg Fe/mL). Both media were diluted with physiological saline by a ratio of 1 : 2. Gd-enhancements T1WI and T2WI were performed for all rabbits at all time points. USPIO-enhancement was sacrificed at 24 h following TBI after Gd-T1WI. The data analyses were performed on the SIEMENS 3.0T Verio MR Graphics work station (Copyright @ SIEMENS AG 2009; series number: 40265; software: NUMARIS/4; version: syngo MRB17; product ID: 097). The sizes of edema were dynamically assessed using T2WI. The blood-brain barrier (BBB) disruption and rehabilitation were assessed by contrast-enhanced T1WI. IR was monitored by Gd-enhanced T1WI before the BBB was repaired and by USPIO-enhanced T1WI after the restoration of BBB.

### 2.4. Pathology Examination

Each group of rabbits was sacrificed for the brain samples immediately when they finished the last scan. The brains were harvested and fixed in 5% formalin. Prior to microscopy examination, gross observations were conducted to detect the primary lesions. The entire brains were incised along the MRI scanning localizer line and cut into 2.0 mm thick slices for pathological section. Following the routine procedures of dehydration, transparency, dewaxing, embedding, paraffin section, and hematoxylin-eosin staining, brain parenchyma was observed under optical microscope (×200; BX50, Olympus, Pangu, Japan). Identification and distribution of inflammatory cells and edema around the injury core were diagnosed by microscopy.

### 2.5. Evaluating Index and Analysis Methods

The severity of edema and IR associated with brain injury were evaluated by sizes measured on T2WI and Gd- or USPIO-enhanced MRI, respectively. The sizes were observed at each time point and measured on the transverse section images perpendicular to traumatic tunnel. The sizes of edema and contrast enhancement were compared between stages in chronological order. Inflammatory cells were discriminated by pathological microscopy. All data were analyzed in SPSS 17.0 statistical software (SPSS, Chicago, IL, USA). Pair sample *t*-test was used for statistical analysis. A Bonferroni corrected *p* < 0.05 was considered statistically significant.

## 3. Result

### 3.1. Dynamic T2WI Presentations of the Edema after TBI

**Figure 1:**
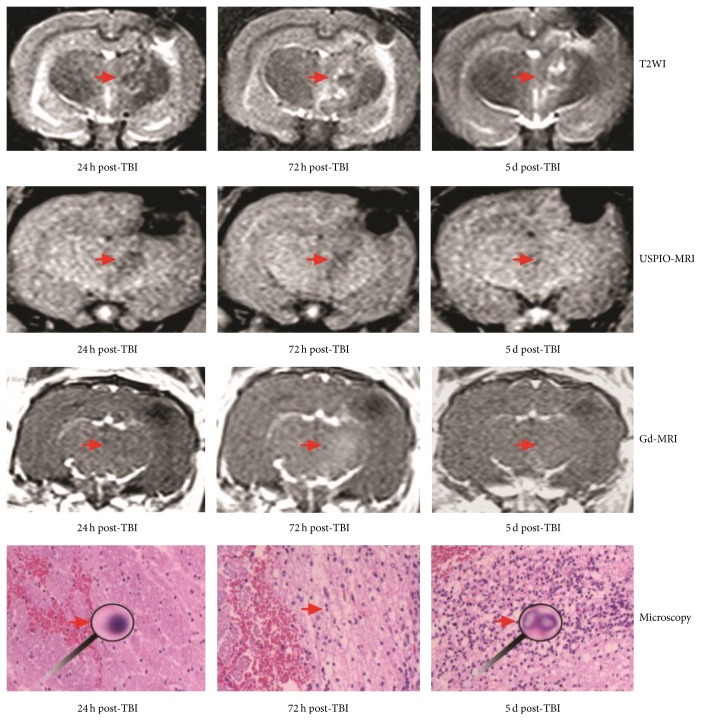
Maps of T2WI, USPIO-MRI, and Gd-MRI were obtained from a same rabbit of the fourth group. The arrows stand for the edema foci.

**Figure 2:**
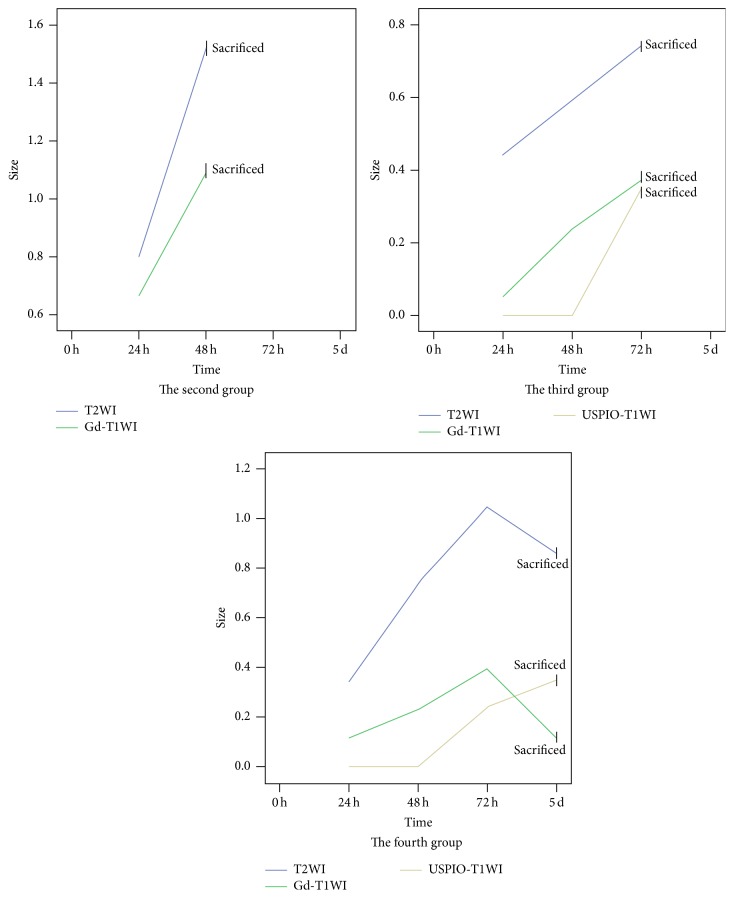
Dynamic changes of sizes of edema following TBI on T2WI, of Gd-enhancement on T1WI and of USPIO-enhancement on T1WI at consecutive monitoring times of different experiment groups.

Twenty experimental mTBI animals successfully conducted the MRI scans. MRI showed that all rabbits were locally injured on the subcortical area of the upper frontal lobe. At the first 24 h after TBI, all rabbits of four groups displayed focal high signal on T2WI. Twenty-four hours later, rabbits of the second, third, and fourth groups demonstrated wider high T2 signal areas. Edema degree in the third and fourth groups had been expected to reach peak in 3 d after TBI and alleviate on 5 d after injury. No significant difference was found between 72 h and 5 d (*p* = 0.25; see Tables 2 and 3 and Figures 1 and 2).

### 3.2. Evolvement of Gd-Enhanced and USPIO-Enhanced MRI Presentations following TBI

At the first 24 h after TBI, only 55% (11/20) lesions could be found on Gd-enhanced T1WI and none was seen on USPIO-enhanced. As progress of lesions, 100% subjects were enhanced by Gd contrast in injured brain regions 48 h after TBI. And the enhanced areas augmented locally on Gd-T1WI in 100% (from the third and fourth group) 72 h after TBI, which were demonstrated through ten rabbits, while 90% lesions were displayed by USPIO-T1WI. Five days after injury, the diminution of the Gd-enhanced sizes was detected on T1WI. Only one rabbit's Gd-enhanced focus area disappeared; however, all five rabbits displayed evident USPIO-enhanced areas larger than those of Gd-enhanced ones (*t* = −2.792, *p* = 0.023) (see Tables 2 and 3 and Figures 1 and 2).

### 3.3. Pathological Findings of IR following TBI in Dynamic Monitoring

The brain tissues of the first group showed that considerable number of leukocytes cells had distributed in the edema regions 24 h after TBI. Inflammatory cells scattered in center to peripheral injury lesions from many to few. Pathological findings of the second group of TBI models showed that slight microglias appeared in dense leukocytes 48 h after injury, but the third group displayed microglias increased 72 h after injury. Specimens of injury brains from the fourth group indicated microglias became the dominant inflammatory cells. Leucocytes were booming to the peak monitored at the 72 h after injury. Five days after injury, the peripheral blood leucocytes diminished with a reverse increase of microglias. At all time points, these two kinds of inflammatory cells constantly distributed in the edema areas (see Figure 1).

## 4. Discussion

A prolonged inflammation state after TBI may last for years and predispose patients to develop other neurological disorders, such as Alzheimer's disease [20]. Multiple inflammatory responses accompanying TBI can worsen and complicate the pathological procedures [15, 20]. TBI patients may endure progressive and persistent impairments in their physical, cognitive, behavioral, and social performance [21]. And hence the inflammatory responses represent potential therapeutic targets. Early monitoring time of inflammatory response is important for the determination of prompt therapeutic strategy.

In this study, IR of the animals were monitored before and after the BBB rehabilitation using tse-fs-T2 and Gd- and USPIO-enhanced T1WI. Lesions were quantitatively analyzed by changes in sizes on tse-fs-T2, Gd-enhanced T1WI, and USPIO-enhanced T1WI maps at every monitoring time. Tissues contusion and vascular tear were the main pathological changes to the injured brain in acute stage, which directly brought about vasogenic interstitial brain edema. Interstitial edema is the main pathophysiological change reflected on T2-based imaging [22].

Twenty four hours after TBI, only a small quantity of peripheral blood leukocytes scattered in the primary foci, and hence, we inferred that edema shown on T2WI was mostly initiated from vascular tear. More than half lesions could be enhanced in Gd-T1WI, which manifested the BBB to be disrupted. The left foci were unenhanced, probably because blood coagulation blocked the teared vascellum or these foci were slightly injured. We found leukocytes rapidly increased and microglias started to appear 48 h after TBI; however, edema area were dramatically enhanced on T2WI maps, and all injured foci were enhanced by Gd-T1WI. In addition, the amount of inflammatory cells, the size of edema, and Gd-enhancement degree reached peak 72 h after injury. Large microglias could be seen with the leukocytes-predominant background. In this stage, IR predominantly attributed to aggravated edema because the tissue contusion and vascular tear had stopped. Moreover, IR caused a breakdown of the BBB, which was verified by rapid extensive Gd-enhanced foci. The edema sizes of the fourth group were restrained 5 days after injury. One case with the smallest injury even disappeared on Gd-T1WI, which implied the disrupted BBB began to be repaired. However, areas of USPIO- enhancement became evident. All sizes of the fourth group were larger than that of Gd-enhanced group. Histological examination found that microglias became the primary inflammatory cells, which were crowded in the edema area.

Secondary to IR injury, the brain was booming quickly after TBI. IR initiated by the impacted tissues [23] further destroyed BBB, which could be reflected in dynamically changed areas of Gd-enhancement [24]. The initial injury resulted in neuronal injury and altered BBB. In the central nervous system, the microglial cells directly reacted to such an injury, and then the activation turned to be chronic one. Microglias could release cytokines and act as antigen presenting cell, which could be distinguished from peripheral macrophage [18].

In the early stage, the post-TBI brain edema might attribute to various vasogenic and/or cytotoxic factors [25]. Antiswelling therapy in TBI patients is still symptomatic treatment (e.g., mannitol infusion, controlled hyperventilation). Vasogenic brain edema was considered as the prevalent edema type following TBI. Actually, IR-induced cytotoxic edema was of decisive pathophysiological importance following TBI because it appeared early during BBB recovery. Unterberg et al. suggested that cytotoxic and vasogenic brain edema could be targeted simultaneously or respectively according to the temporal prevalence in TBI [25]. Neurogenic inflammation might play an integral role in the development of edema following TBI [26]. Our findings demonstrated that the spatial patterns of inflammatory cells distribution and edema appearance, which were consistent with the pathologies. Vasogenic edema disappeared, when disrupted BBB was recovered. T2WI clearly displayed the edema regions, which indirectly reflected the spreading of inflammatory cell population and the severity of IR. These findings implied that the accurate diagnosis of the severity and prognosis of TBI could be based on analyses of the post-TBI IR by multimodality MRI.

In this study, we improved the animal model of mild TBI built by Turtzo et al. [11]. In their study, a mechanical TBI model of controlled impact injury was developed in the unilateral motor cortex of craniotomied rabbits by a 5 mm impactor tip. Since preliminary experiment of this study demonstrated the MRI maps could not clearly display the injury lesion in the cortex due to skull magnetic and chemical shift artifacts, a sterile impactor needle pricking was used to produce a mechanical injury located at the deep cerebellar subcortex.

USPIO enabled more sensitive assessment of leukocyte (mainly macrophage) infiltration by the MR technologies independent from a disturbance of the BBB [27]. Combination of Gd- and USPIO-enhancement MRI might noninvasively monitor the dynamic IR following TBI in time. But an inherent limitation of USPIO was that iron oxide particles were consumed by various macrophages except stem cells. USPIO could not directly detect the information of cellular survival or death, proliferation, and differentiation, which might provide error messages for imaging [28]. Recently, there were some other intracellular contrast agents, such as [^(11)^C] (R)-PK11195-PET, [^(18)^F] fluoroethyl-DAA 1106-PET, and [^(18)^F] fluorodeoxyglucose- PET [29], which might be used as molecular tracers to monitor long-term response and changes of brain structure and function. However, there were difficulties for these tracer agents shifting into routine clinical usage. Clear display of lesions near to cranium was affected due to magnetic artifacts. Further studies are urgently needed for accurately monitoring IR following TBI for precise treatment and improving the prognosis.

## 5. Conclusions

In this study, IR following TBI before and after the rehabilitation of the blood-brain barrier (BBB) in rabbits was accessed via both USPIO-enhanced and Gd-enhanced MRI. The edema areas of the lesions sharply reached peak 72 h after injury and became alleviated five days after TBI. Peripheral blood leucocytes were the major contributor of the edema before the BBB rehabilitation, which was indirectly shown by Gd-enhanced T1WI. USPIO-enhancement turned into predominance regardless of the BBB rehabilitation. These findings supported that IR following injury was dynamically evolved in a distinct mode before and after the BBB rehabilitation, which could be monitored by the combination of Gd-MRI and USPIO-MRI.

## Figures and Tables

**Table 1:** The protocols of MR sequences.

Sequence	t1-fl2d-tra	t2-tse-tra	USPIO-t2-tra	Gd-t1-tra
FOV read (mm)	200	200	200	200
FOV phase	100%	100%	100%	100%
Slice thickness (mm)	2	2	2	2
TR (ms)	250	6000	6000	250
TE (ms)	2.48	116	116	2.48
Averages	2	2	2	2
Disc factor	30%	30%	30%	30%
Phase encode direction	R → L	R → L	R → L	R → L
Phase over sampling	30%	30%	30%	30%
Slice over sampling	—	—	—	—
Slice per slab	—	—	—	—
Flip angle	70%	150%	150%	70%
Fat suppression	No	Yes	Yes	Yes
MTC	No	No	No	No

**Table 2:** Changes of sizes of edema and contrast-enhancement during dynamic monitoring.

Number of groups	Number of rabbits	24 h after TBI			48 h after TBI			72 h after TBI			120 h after TBI		
		T2WI	Gd-T1WI	USPIO-T1WI	T2WI	Gd-T1WI	USPIO-T1WI	T2WI	Gd-T1WI	USPIO-T1WI	T2WI	Gd-T1WI	USPIO-T1WI
1	1	0.16	0	0	—	—	—	—	—	—	—	—	—
	2	0.25	0	0	—	—	—	—	—	—	—	—	—
	3	0.22	0.15	0	—	—	—	—	—	—	—	—	—
	4	0.2	0	0	—	—	—	—	—	—	—	—	—
	5	0.23	0.21	0	—	—	—	—	—	—	—	—	—

2	1	1.6	1.6	0	3.3	2.3	0	—	—	—	—	—	—
	2	0.55	0.5	0	1.04	0.68	0	—	—	—	—	—	—
	3	0.6	0.43	0	0.74	0.52	0	—	—	—	—	—	—
	4	0.16	0	0	0.16	0.1	0	—	—	—	—	—	—
	5	1.1	0.8	0	2.4	1.86	0	—	—	—	—	—	—

3	1	0.22	0	0	0.34	0.2	0	0.4	0.15	0.16	—	—	—
	2	0.46	0	0	0.59	0.4	0	0.82	0.46	0.32	—	—	—
	3	0.05	0	0	0.08	0.08	0	0.17	0.17	0.2	—	—	—
	4	1	0.26	0	1	0.21	0	1.3	0.3	0.28	—	—	—
	5	0.48	0	0	0.96	0.3	0	1.02	0.78	0.8	—	—	—

4	1	1	0.26	0	1.2	0.39	0	1.7	0.6	0.37	1.1	0.21	0.56
	2	0.1	0.1	0	0.16	0.1	0	0.16	0.12	0	0	0	0.13
	3	0.25	0.12	0	0.81	0.3	0	1.02	0.6	0.22	1.3	0.12	0.3
	4	0.25	0.12	0	0.81	0.21	0	1.62	0.41	0.4	1.3	0.19	0.46
	5	0.12	0	0	0.72	0.16	0	0.75	0.25	0.22	0.59	0.06	0.3

**Table 3:** Paired sample test of dynamic monitoring time.

	T2WI			Gd-T1WI			USPIO-T1WI		
	24 h–48 h	48 h–72 h	72 h–5 d	24 h–48 h	48 h–72 h	72 h–5 d	24 h–48 h	48 h–72 h	72 h–5 d
*t*	−3.324	−2.284	1.345	−3.23	−3.061	4.188	—	—	−4.588
*p*	0.005	0.019	0.25	0.006	0.014	0.014	—	—	0.01
